# *Quercus persica* lozenges versus placebo for reducing gag reflex severity in adult dental patients: a randomized double-blind controlled trial

**DOI:** 10.1007/s44445-026-00207-2

**Published:** 2026-07-20

**Authors:** Atefeh Mohammadi, Mohsen Taghizadeh, Reza Hosseiniara, Hossien Akbari, Ameneh Taghdisi Kashani

**Affiliations:** Department of Pediatrics, School of Dentistry, Kashan University of Medical Sciences, Kashan, Islamic Republic of Iran

**Keywords:** Gag reflex, *Quercus persica*, Persian oak, Lozenge, Herbal medicine

## Abstract

The gag reflex is a common challenge during dental procedures, often impairing clinical performance and causing patient discomfort. While pharmacological interventions are commonly employed, they can lead to adverse effects or be poorly tolerated. Herbal alternatives with local mucosal activity have gained increasing attention as potentially safer adjuncts. *Quercus persica* (Persian oak), rich in tannins, exhibits astringent and mucosal-desensitizing properties that may contribute to reducing gag reflex sensitivity. To evaluate the efficacy of a Quercus persica extract lozenge in reducing gag reflex severity in adult dental patients. In this randomized, double-blind, placebo-controlled clinical trial, 80 adult patients with a documented history of gag reflex were enrolled and equally allocated (1:1) into intervention and placebo groups (n = 40 each). Participants received either a 1-gram lozenge containing 200 mg of Quercus persica extract or an identical placebo. Gag reflex severity was assessed before and after intervention using a standardized 4-point ordinal scale across five intraoral regions. The primary outcome was the change in overall gag reflex score. Baseline demographic characteristics were comparable between the two groups. The Quercus persica group exhibited a significant reduction in overall gag reflex score (from 5.98 ± 2.26 to 1.38 ± 1.72; p = 0.001), in the placebo group (from 5.83 ± 2.01 to 5.10 ± 2.10; p = 0.002). Between-group comparison confirmed a significantly greater reduction in the intervention group (p < 0.001). Region-specific analysis revealed the greatest improvements in the soft palate and tonsillar regions, with mean reductions of 1.75 and 1.73 points, respectively (p < 0.001). Quercus persica lozenges were associated with a statistically significant reduction in gag reflex severity compared to placebo, particularly in highly sensitive intraoral regions. These findings suggest that Quercus persica may serve as a potential adjunctive approach for gag reflex management; however, conclusions should be interpreted with caution given the short-term assessment, and further studies are required to evaluate long-term efficacy, safety, and patient-reported outcomes.

## Introduction

The gag reflex, also known as the pharyngeal reflex, is a protective neuromuscular response primarily mediated by cranial nerves IX (glossopharyngeal) and X (vagus), and is typically elicited by tactile or psychological stimulation of the oropharyngeal region. While this reflex serves as a physiological defense mechanism to prevent the aspiration of foreign objects, an overactive gag reflex can pose significant challenges during routine dental and medical procedures (Nikkerdar et al. 2024). Common interventions, such as intraoral radiography, impression-taking, and restorative treatments, often elicit this reflex, leading to patient discomfort, interruptions in care, and diminished cooperation. In more severe cases, patients may develop avoidance behaviors toward dental care, which can adversely impact their oral and overall health (Hekmatian et al. 2012; Okamoto et al. 2024).

Research indicates that approximately 8.2% of dental patients experience nausea and gagging during procedures (Okamoto et al. 2024), while higher prevalence rates (up to 44%) have been reported among individuals undergoing prosthodontic procedures or dental impressions (Elham et al. 2020; Torabi et al. 2024). The psychological repercussions of repeated gagging can be profound, contributing to anxiety, distress, and reluctance to seek dental care. Collectively, these factors highlight the need for effective, safe, and clinically practical interventions capable of minimizing gag reflex severity without increasing treatment complexity or patient burden.

Over the years, several strategies have been proposed to alleviate the gag reflex. Behavioral techniques, such as distraction, controlled breathing, and hypnotherapy, have shown variable efficacy and often necessitate trained personnel and active patient cooperation (Mehdizadeh et al. 2023). Additionally, acupuncture and acupressure have been explored as alternative interventions, with some studies reporting favorable outcomes in reducing gagging during impression-taking or upper arch procedures (Lu et al. 2000; Usichenko et al. 2020). However, the routine clinical applicability of these approaches remains limited due to variability in effectiveness, time requirements, and practitioner expertise.

Pharmacological approaches, including local anesthetics (e.g., lidocaine spray), anxiolytics, and sedatives (e.g., diazepam or nitrous oxide), have been widely utilized to manage exaggerated gag reflexes (Eachempati et al. 2019; Torabi et al. 2024). Although these agents can be effective, they are not without potential drawbacks, such as systemic side effects, allergic reactions, or prolonged recovery times, particularly in elderly or medically compromised patients. Moreover, topical anesthetics may alter normal oral sensation, which can negatively affect patient comfort and procedural tolerance.

In light of these challenges, there has been growing interest in plant-based and herbal interventions with astringent, anesthetic, or anti-inflammatory properties. Among the most promising natural agents are tannin-rich herbal compounds, known for their ability to desensitize mucosal surfaces and reduce hypersensitivity responses. Tannins exert their effects by binding to proteins and forming complexes that reduce mucosal permeability and reactivity (Deryabin and Tolmacheva 2015). This localized biochemical interaction may contribute to reduced stimulation of sensory receptors involved in the gag reflex.

One notable tannin-rich plant is the Persian oak (*Quercus persica*), a species indigenous to the Zagros region of Iran. Oaks (*Quercus* spp.) have been extensively investigated for their rich phytochemical profiles and therapeutic potential, including antimicrobial, antioxidant, anti-inflammatory, and wound-healing properties (Akram and Masoud 2012; Azizi et al. 2014; Banc et al. 2023). The galls, bark, and seeds of various oak species exhibit significant biological activity across multiple domains, including oral health.

Previous research has demonstrated that oak extracts possess antimicrobial activity against oral pathogens such as *Streptococcus mutans*, supporting their potential role in oral health management (Basri et al. 2011; Mohammadi-Sichani et al. 2015). Additionally, polyphenols derived from oak, including ellagic acid, gallic acid, and flavonoids, contribute to their anti-inflammatory and mucosal-protective effects, thereby supporting tissue healing and modulating sensory responses in the oral mucosa (Castillo-Mendoza et al. 2022; Coman et al. 2024).

The use of lozenge-based delivery systems has gained attention due to their ease of administration and ability to provide prolonged mucosal contact. Lozenges facilitate sustained release of active compounds, thereby enhancing localized therapeutic effects. Previous studies have evaluated lozenges containing herbal or pharmacological agents for oral conditions such as ulcers and sore throat, with limited but emerging evidence suggesting potential benefits in reducing gag reflex sensitivity (Hekmatian et al. 2012; Torabi et al. 2024).

Recent investigations into oak-derived extracts, particularly from bark and galls, have highlighted their role in enhancing mucosal integrity and reducing hypersensitivity. Preliminary findings suggest that these compounds may contribute to lowering gag reflex thresholds; however, direct clinical evidence in dental settings remains scarce (Dardmah and Farahpour 2021; Kim et al. 2024). Furthermore, although commercial oak-derived products (e.g., Robuvit^®^) have demonstrated safety in other clinical contexts, their applicability to oral reflex modulation has not been established (Custódio et al. 2013; Oliveira et al. 2023).

Despite these promising findings, the specific application of *Quercus persica* extract for managing the gag reflex in dental patients remains insufficiently investigated. Existing studies on tannin-rich or astringent agents (e.g., *Punica granatum*, *Elaeagnus angustifolia*) report modest and variable outcomes, highlighting the need for more robust clinical evidence (Hekmatian et al. 2012; Elham et al. 2020; Nikkerdar et al. 2024). Accordingly, well-designed randomized controlled trials are required to determine the efficacy and clinical relevance of such interventions.

Therefore, the present study was designed to evaluate, under controlled clinical conditions, the effect of *Quercus persica* extract lozenges on gag reflex severity in adult dental patients with a history of gagging.

## Methods

### Study design

This randomized, double-blind, placebo-controlled clinical trial was conducted in 2020 at the Radiology Department of the School of Dentistry, Kashan University of Medical Sciences, Kashan, Iran.

### Ethical considerations

The investigational Quercus persica lozenge used in this study was a locally prepared research product developed exclusively for this clinical trial and was not a commercially marketed medicinal product. Therefore, approval by the United States Food and Drug Administration (FDA) was not applicable.

The study was conducted in accordance with national ethical and regulatory requirements, the Declaration of Helsinki, and Good Clinical Practice (GCP) guidelines. The study protocol was approved by the Research Ethics Committee of Kashan University of Medical Sciences (IR.KAUMS.MEDNT.REC.1399.048), and the trial was prospectively registered in the Iranian Registry of Clinical Trials (IRCT20130211012438N33) before participant enrollment.

Prior to enrollment, all participants received verbal and written information regarding the study objectives, procedures, expected duration, potential benefits and risks, confidentiality of personal information, and their right to withdraw from the study at any time without affecting their access to dental care. Participants were given adequate opportunity to ask questions before providing written informed consent.

Participation was entirely voluntary, and all personal information was handled confidentially. No participant’s access to dental treatment was influenced by their decision to participate in or withdraw from the study.

### Participants

Eligible participants were adults aged ≥ 18 years with a documented history of gag reflex during previous dental procedures. Inclusion criteria were: (1) requirement for dental radiographic imaging, (2) absence of systemic diseases, and (3) absence of oral or sensory lesions. Exclusion criteria included: (1) diagnosed central or peripheral neurological disorders, (2) active oral, respiratory, or gastrointestinal diseases, (3) current use of medications affecting neuromuscular or sensory responses, (4) known allergies to herbal products, and (5) unwillingness to provide informed consent.

### Sample size

Sample size calculation was based on a previous study evaluating the effect of *Elaeagnus angustifolia* on gag reflex reduction (Hekmatian et al. 2012). Assuming a large effect size, a two-sided alpha level of 0.05, and statistical power of 90%, the minimum required sample size was calculated. To compensate for potential dropouts, the total sample size was increased to 80 participants (40 per group).

### Randomization and allocation concealment

Participants were randomly assigned in a 1:1 ratio to either the intervention group (*Quercus persica* lozenge) or the placebo group using a computer-generated randomization sequence (block randomization method with equal block sizes). Allocation concealment was ensured using sequentially numbered, opaque, sealed envelopes prepared by an independent laboratory technician not involved in recruitment, intervention, or outcome assessment.

### Blinding

Blinding was maintained for participants, clinicians administering the intervention, outcome assessors, and data analysts. Lozenges were individually packaged and coded to mask their identity. The active and placebo lozenges were identical in shape, size, color, texture, and packaging. The blinding code was not broken until completion of data analysis.

### Intervention

#### Oak extract preparation

*Quercus persica* seeds were procured from certified vendors in the Zagros region (Sanandaj, Iran) and authenticated by a qualified botanist. A voucher specimen was retained for reference. The seeds were dried at 60 °C, ground into a fine powder, and then macerated in a 70% hydroalcoholic solution at 70 °C for 72 h. The extract was filtered and concentrated by drying at 50 °C. All procedures were conducted under controlled hygienic conditions at the Research Center for Biochemistry and Nutrition in Metabolic Diseases, Kashan University of Medical Sciences.

#### Lozenge formulation

To prepare the oak lozenges, 80 g of sugar were dissolved in heated water until a syrup-like consistency was achieved, followed by the addition of 20 g of oak extract, yielding a final dry weight of 100 g. The mixture was molded into lozenges weighing approximately 1 g each, with each lozenge containing 200 mg of *Quercus persica* extract. Participants were instructed to slowly dissolve the lozenge in the oral cavity for approximately 3–5 min prior to the procedure, without chewing or swallowing it whole.

#### Placebo formulation

Placebo lozenges were prepared using the same base formulation without the active extract. These lozenges were identical in appearance, weight, color, and texture. Both active and placebo lozenges were produced under the supervision of the research team and packaged in coded wrappers to maintain blinding.

### Outcome assessment and standardization

#### Operator calibration

All gag reflex assessments were performed by a single trained clinician to minimize inter-examiner variability. Prior to the study, the examiner underwent calibration sessions using pilot cases to ensure consistency in stimulation technique and scoring. Intra-examiner reliability was assessed and deemed acceptable.

#### Assessment procedure

All assessments were conducted under standardized clinical conditions, with patients seated in an upright dental chair position and using identical examination instruments. The gag reflex was evaluated immediately before and 5 min after lozenge administration.

#### Standardized scoring system

The gag reflex was assessed using a 4-point ordinal scale across five intraoral regions: soft palate, tonsillar pillars, hard palate, sublingual area, and visual observation. Gentle and standardized tactile stimulation was applied to each anatomical region using a dental mirror or probe in a consistent sequence.

Each region was scored independently based on the observed severity of the gag reflex (Table 1). This scale was adapted from validated tools, including the Gag Trigger Point Index (GTPI) and previously used scoring systems (Hekmatian et al. 2012; Nikkerdar et al. 2024; Torabi et al. 2024).

#### Outcome measures

The primary outcome of this study was the change in total gag reflex score, assessed at baseline and five minutes after intervention to capture the immediate local effect of the lozenge. Regional gag scores were evaluated across five intraoral regions -visual observation, sublingual region, hard palate, soft palate, and tonsillar region- using a scale from 0 to 3, reflecting increasing severity of the reflex. The total gag reflex score was calculated as the sum of these regional scores, resulting in a possible range from 0 to 15. To quantify the effect of the intervention, a reflex reduction score was calculated for each participant by subtracting the post-treatment score from the baseline score. Higher baseline scores indicated greater initial gag reflex severity, whereas larger reduction scores reflected a stronger therapeutic effect. This approach facilitated both within-subject comparisons over time and between-group analyses, providing a comprehensive evaluation of Quercus persica’s efficacy across multiple oral regions. All assessments were conducted by a single calibrated examiner to ensure consistency in timing and minimize measurement bias.

### Statistical analysis

All analyses were performed using SPSS version 21 (IBM Corp., Armonk, NY, USA). Continuous variables, including regional and total gag reflex scores, were summarized as means ± standard deviations (SD), while categorical variables, such as sex distribution, were reported as frequencies and percentages. The normality of continuous data was assessed with the Shapiro–Wilk test, revealing that most outcome variables were non-normally distributed. Given the ordinal nature of the gag reflex scores and the observed non-normal distribution, non-parametric statistical methods were applied for all analyses. Within-group comparisons between baseline and five minutes post-intervention were performed using the Wilcoxon signed-rank test, appropriate for paired ordinal or non-normally distributed data. Between-group comparisons of reduction scores (calculated as baseline minus post-treatment values) were conducted using the Mann–Whitney U test, suitable for independent, non-parametric datasets. Categorical variables, including sex distribution, were analyzed using Chi-square or Fisher’s exact tests, depending on expected frequencies. For any paired categorical outcomes, McNemar’s test would have been applied; however, no such outcomes were included in this study. All statistical tests were two-sided, and a p-value < 0.05 was considered statistically significant. The timing of post-treatment assessments was deliberately set at five minutes after lozenge administration to reflect the peak local effect of the Quercus persica extract.

**Table 1:** Gag reflex severity scores and descriptions

Score	Description
0	No reflex – No gag response upon stimulation or visual inspection
1	Mild reflex – Gag response only to high-sensitivity areas (e.g., soft palate, tonsillar area)
2	Moderate reflex – Gag response triggered by low-sensitivity areas (e.g., hard palate, sublingual area)
3	Severe reflex – Reflex triggered by visual stimulus alone or anticipatory response before contact

## Results

**Fig. 1:**
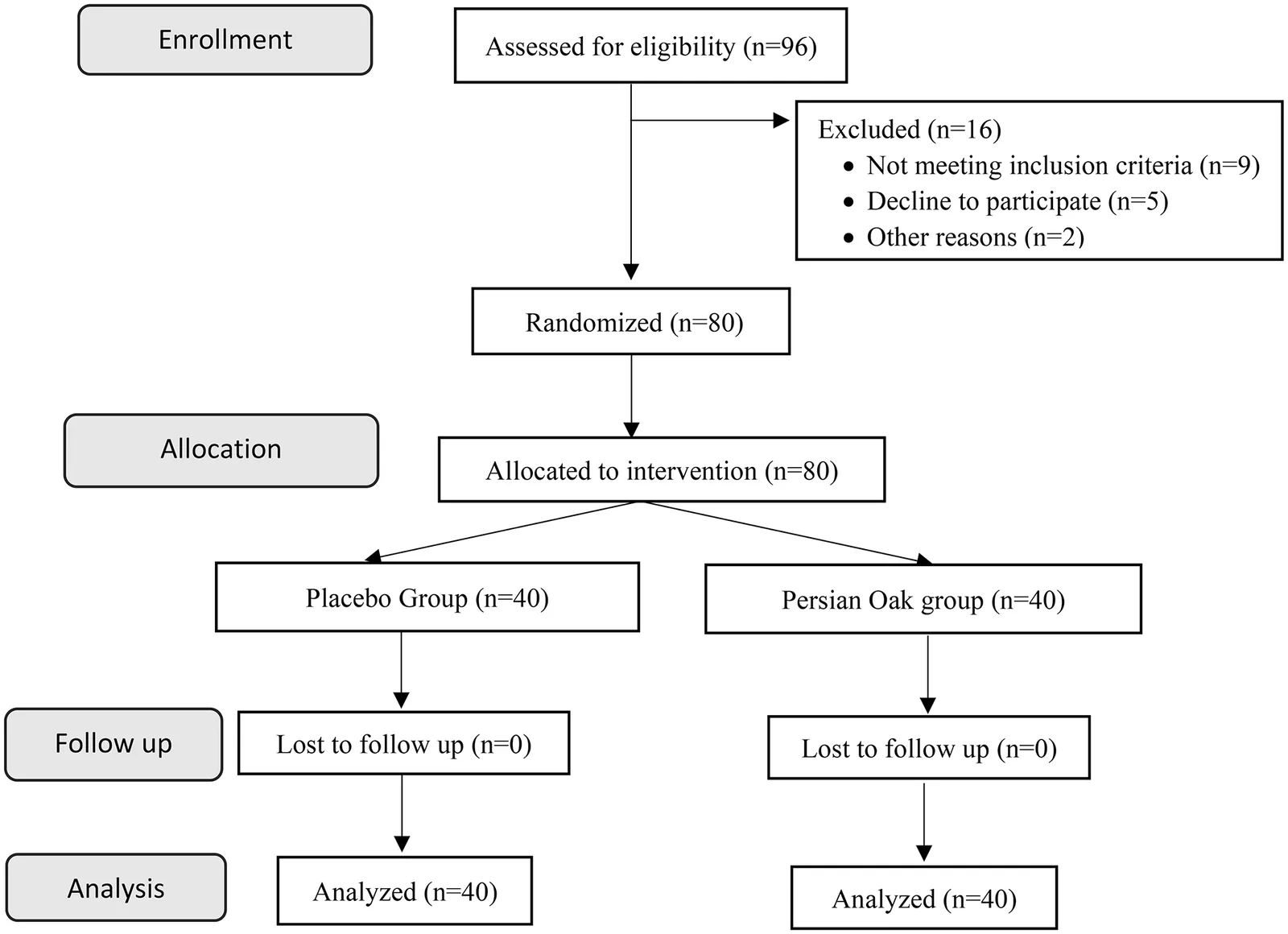
Consort flowchart for participant’s selection

A total of 80 adult participants were enrolled and randomly assigned into two intervention groups, with 40 participants allocated to the Quercus persica (Persian oak) group and 40 to the placebo group. Baseline demographic characteristics were well balanced between groups. In the placebo group, 35 participants (87.5%) were female and 5 (12.5%) were male, whereas in the Persian oak group, 34 participants (85%) were female and 6 (15%) were male. The mean ages were virtually identical, 27.60 ± 6.25 years in the placebo group and 27.58 ± 6.15 years in the intervention group (*p* = 0.986). These findings indicate that randomization achieved effective demographic matching, minimizing potential confounding effects related to age or sex (Table 2). All participants completed the study, with no losses to follow-up, ensuring a complete dataset for analysis. The CONSORT flow diagram depicts participant enrollment, allocation, follow-up, and analysis (Fig. 1).

**Table 2:** Baseline characteristics of participants in the Persian oak and placebo groups

Variable	Category	Placebo group (*n* = 40)	Persian oak group (*n* = 40)	*P*-value
Sex, n (%)	Female	35 (87.5%)	34 (85.0%)	0.745
	Male	5 (12.5%)	6 (15.0%)	
Age (years)	Mean ± SD	27.60 ± 6.25	27.58 ± 6.15	0.986

### Changes in gag reflex scores before and after intervention

Gag reflex severity was assessed five minutes after lozenge administration across five intraoral regions as well as through visual observation, with mean scores summarized in Table 3. Within-group analysis demonstrated that participants receiving the Persian oak lozenge experienced statistically significant reductions in gag reflex scores in all regions. Specifically, scores decreased in the visual observation category from 0.20 ± 0.51 to 0.08 ± 0.35 (*p* = 0.023), in the sublingual region from 0.68 ± 1.05 to 0.13 ± 0.40 (*p* < 0.001), in the hard palate from 0.53 ± 0.88 to 0.08 ± 0.27 (*p* < 0.001), in the soft palate from 2.28 ± 0.50 to 0.53 ± 0.64 (*p* < 0.001), and in the tonsillar region from 2.30 ± 0.46 to 0.57 ± 0.71 (*p* < 0.001). The overall gag score similarly decreased from 5.98 ± 2.26 to 1.38 ± 1.72 (*p* < 0.001).

In contrast, the placebo group showed smaller and region-specific reductions, with no statistically significant change in visual observation (0.05 ± 0.32 to 0; *p* = 0.323), while minor but significant decreases were observed in the sublingual region (0.57 ± 0.90 to 0.48 ± 0.81; *p* = 0.044), hard palate (0.65 ± 1.10 to 0.53 ± 0.96; *p* = 0.023), soft palate (2.30 ± 0.46 to 2.03 ± 0.83; *p* = 0.003), tonsillar region (2.25 ± 0.44 to 2.08 ± 0.57; *p* = 0.006), and overall gag score (5.83 ± 2.01 to 5.10 ± 2.10; *p* = 0.002). These results indicate that the therapeutic effect of Quercus persica is both robust and consistent across multiple intraoral regions, particularly in areas with heightened sensitivity.

**Table 3:** Mean gag reflex scores before and after intervention

Intraoral Region	Group	Pre-treatment (Mean ± SD)	Post-treatment (Mean ± SD)	*P*-value (within-group)
Visual Observation	Placebo	0.05 ± 0.32	0.00 ± 0.00	0.323
	Persian oak	0.20 ± 0.51	0.08 ± 0.35	0.023
Sublingual Region	Placebo	0.57 ± 0.90	0.48 ± 0.81	0.044
	Persian oak	0.68 ± 1.05	0.13 ± 0.40	0.001
Hard Palate	Placebo	0.65 ± 1.10	0.53 ± 0.96	0.023
	Persian oak	0.53 ± 0.88	0.08 ± 0.27	0.001
Soft Palate	Placebo	2.30 ± 0.46	2.03 ± 0.83	0.003
	Persian oak	2.28 ± 0.50	0.53 ± 0.64	0.001
Tonsillar Region	Placebo	2.25 ± 0.44	2.08 ± 0.57	0.006
	Persian oak	2.30 ± 0.46	0.57 ± 0.71	0.001
Overall Gag Score	Placebo	5.83 ± 2.01	5.10 ± 2.10	0.002
	Persian oak	5.98 ± 2.26	1.38 ± 1.72	0.001

### Between-group comparison of gag reflex reduction

Reductions were calculated as baseline minus post-treatment scores (five minutes post-lozenge). As summarized in Table 4, participants in the Persian oak group experienced significantly greater reductions compared to the placebo group across most regions. The sublingual region exhibited a mean reduction of 0.55 ± 0.81 points in the oak group versus 0.10 ± 0.30 in the placebo group (*p* = 0.002). In the hard palate, mean reductions were 0.45 ± 0.71 versus 0.13 ± 0.33 (*p* = 0.011), and in the soft palate 1.75 ± 0.44 versus 0.28 ± 0.55 (*p* < 0.001). The tonsillar region showed a reduction of 1.73 ± 0.51 in the intervention group versus 0.18 ± 0.38 in the placebo group (*p* < 0.001). Overall gag scores decreased by 4.60 ± 1.39 points in the oak group compared to 0.73 ± 1.38 points in the placebo group (*p* < 0.001). The visual observation category showed a smaller between-group difference (0.13 ± 0.33 versus 0.05 ± 0.32; *p* = 0.306), suggesting that anticipatory gag responses may be less sensitive to single-dose intervention but still trend toward improvement with Quercus persica.

**Table 4:** Reduction in mean gag reflex scores (post-treatment – baseline)

Intraoral Region	Placebo group (Mean ± SD)	Persian oak group (Mean ± SD)	*P*-value (between-group)
Visual Observation	0.05 ± 0.32	0.13 ± 0.33	0.306
Sublingual Region	0.10 ± 0.30	0.55 ± 0.81	0.002
Hard Palate	0.13 ± 0.33	0.45 ± 0.71	0.011
Soft Palate	0.28 ± 0.55	1.75 ± 0.44	0.001
Tonsillar Region	0.18 ± 0.38	1.73 ± 0.51	0.001
Overall Gag Score	0.73 ± 1.38	4.60 ± 1.39	0.001

### Overall gag reflex score

The overall gag reflex score decreased markedly in the Persian oak group from 5.98 ± 2.26 at baseline to 1.38 ± 1.72 five minutes post-lozenge, compared to a modest decrease in the placebo group from 5.83 ± 2.01 to 5.10 ± 2.10. The mean reduction of 4.60 ± 1.39 in the intervention group was highly significant relative to placebo (0.73 ± 1.38; *p* < 0.001).

### Safety and tolerability

No adverse events, complications, or side effects were reported in either group throughout the study period, indicating that a single dose of the Persian oak lozenge is well tolerated and safe for adult dental patients.

## Discussion

This randomized, double-blind, placebo-controlled clinical trial investigated the effects of Quercus persica (Persian oak) lozenges on the severity of the gag reflex in adult dental patients, a common challenge that can compromise both patient comfort and procedural efficiency. Administration of a single lozenge produced a statistically and clinically meaningful reduction in gag reflex scores across multiple intraoral regions, suggesting its potential as a locally acting, well-tolerated intervention for patients with heightened reflex sensitivity (Hekmatian et al. 2012; Nikkerdar et al. 2024; Torabi et al. 2024). These findings are particularly relevant in light of the limitations associated with conventional pharmacological approaches, which, although effective, are often associated with undesirable side effects, patient discomfort, and reduced compliance.

The observed reduction in gag reflex severity can be plausibly explained by the phytochemical properties of Quercus persica. The oak extract is rich in tannins, particularly tannic acid, a polyphenol with well-documented astringent, anti-inflammatory, and mucosal-protective properties (Deryabin and Tolmacheva 2015; Şöhretoğlu and Renda 2020). Tannins form complexes with salivary and epithelial proteins, creating a localized barrier that stabilizes mucosal cell membranes and protein receptors. This mucosal stabilization likely diminishes the sensitivity of mechanoreceptors and chemoreceptors responsible for triggering the gag reflex, particularly in anatomically sensitive regions such as the soft palate and tonsillar pillars. These areas are well-recognized as the most reflexogenic sites in the oral cavity, which may explain the particularly pronounced reductions observed in these regions in our study (Elham et al. 2020; Şöhretoğlu and Renda 2020).

Beyond tannins, Quercus persica contains other bioactive compounds, including gallic acid, ellagic acid, and flavonoids, all of which have demonstrated anti-inflammatory and antioxidant effects in preclinical studies (Banc et al. 2023; Coman et al. 2024). By mitigating local inflammation and oxidative stress, these compounds may reduce mucosal hypersensitivity and the likelihood of exaggerated reflex responses. The cumulative effect of these phytochemicals may account for the broad and consistent reduction in gag reflex severity across all five assessed intraoral regions, a pattern that distinguishes this intervention from other herbal remedies that often exhibit more region-specific effects (Anlas et al. 2019; Dardmah and Farahpour 2021). These mechanistic insights underscore the biological plausibility of the intervention and provide a rational basis for its observed efficacy.

Our findings align with previous research examining tannin-rich herbal interventions. Nikkerdar and colleagues demonstrated that a tannic acid-based mucoadhesive gel could reduce gag reflex severity, highlighting the feasibility of localized polyphenol administration as a therapeutic strategy (Nikkerdar et al. 2024). Similarly, other herbal agents, including Elaeagnus angustifolia and peppermint oil, have demonstrated efficacy, although typically in limited or region-specific contexts (Hekmatian et al. 2012; Okamoto et al. 2024). In contrast, the current study shows that Quercus persica lozenges produced significant reductions in gag reflex scores across all intraoral regions, suggesting a more generalized desensitizing effect. This difference may be attributable to the lozenge formulation, which ensures prolonged contact with the mucosal surface, enhancing the local pharmacological action without systemic absorption. The ease of administration and the fact that no specialized clinical training is required further enhance the clinical utility of this intervention, making it a potentially attractive option for routine dental practice.

Compared to conventional pharmacological agents such as lidocaine sprays or benzydamine hydrochloride, *Quercus persica* lozenges offer distinct advantages. Although synthetic agents are effective, their use is frequently associated with mucosal numbness, unpleasant taste, hypersensitivity reactions, and patient discomfort, all of which can limit acceptance and adherence (Torabi et al. 2024). In contrast, the oak lozenge in this trial was well tolerated, with no adverse events reported, suggesting a favorable safety profile. However, it is important to note that no direct comparisons with standard pharmacological treatments were performed in this study, and therefore, while promising, claims regarding superior tolerability remain speculative and warrant further investigation. Nevertheless, the absence of systemic effects or sedation implies that this intervention could be especially suitable for patients in whom traditional pharmacological sedatives are contraindicated, such as those with significant comorbidities, the elderly, or individuals with polypharmacy concerns (Eachempati et al. 2019; Torabi et al. 2024).

Although no drug interactions were observed in this study, it is important to consider potential interactions in clinical practice. The phytochemical composition of Persian oak, including tannins and flavonoids, could theoretically influence the absorption or metabolism of concomitantly administered medications. Tannins may bind proteins and metal ions, potentially reducing the oral bioavailability of certain drugs, while flavonoids may modulate cytochrome P450 enzyme activity, affecting hepatic metabolism of medications with a narrow therapeutic window. While the lozenge acts primarily locally with minimal systemic absorption, clinicians should exercise caution in patients on multiple medications, and future studies are warranted to systematically evaluate the safety profile of Persian oak in polypharmacy contexts (Hu et al. 2005; Posadzki et al. 2013).

A particularly interesting finding was the improvement observed in the visual observation category, which measures anticipatory gagging occurring prior to physical stimulation. Patients receiving Quercus persica lozenges exhibited a statistically significant reduction in this measure (*p* = 0.023), whereas the placebo group showed no change. This effect suggests that the lozenge may exert neuromodulatory or anxiolytic effects, potentially attenuating anticipatory or psychologically mediated components of the gag reflex. While speculative, this finding raises intriguing questions regarding the potential interaction between mucosal desensitization and central sensory perception, warranting future studies that integrate psychological and physiological assessments to explore these mechanisms more thoroughly (Mehdizadeh et al. 2023; Okamoto et al. 2024).

The study’s methodological rigor further supports the validity of these findings. Randomization was computer-generated, allocation was concealed, and both participants and investigators were blinded, minimizing potential bias. Groups were balanced in age (*p* = 0.986) and sex (*p* = 0.745), and intraoral assessments were conducted by a calibrated examiner (kappa = 0.89) using a standardized scoring system, ensuring reliability across multiple regions. Furthermore, statistical analyses were conducted using non-parametric methods appropriate for ordinal data, reinforcing the robustness of the reported p-values (Hekmatian et al. 2012; Eachempati et al. 2019; Nikkerdar et al. 2024; Torabi et al. 2024). This combination of rigorous design, objective measurement, and appropriate statistical methodology lends confidence to the observed treatment effects.

Despite these strengths, several limitations must be acknowledged. The study evaluated only the short-term effect of a single lozenge, leaving the duration of benefit and the optimal dosing schedule unclear. The sample consisted solely of healthy young adults, which limits generalizability to pediatric, geriatric, or medically compromised populations, who may differ in mucosal sensitivity, reflex thresholds, or pharmacokinetic responses. Additionally, patient-reported outcomes, such as taste acceptability, ease of use, or preference relative to other anti-gag strategies, were not collected, which limits the assessment of real-world applicability. The study also did not compare the oak lozenge with standard pharmacological agents, precluding direct conclusions about relative efficacy or tolerability. While the slight improvement in the placebo group suggests a minor psychological contribution, the magnitude and consistency of the effects in the oak group point to a pharmacologically mediated mechanism, though this remains to be confirmed through mechanistic studies, such as assessments of mucosal protein binding or sensory nerve activity (Elham et al. 2020; Şöhretoğlu and Renda 2020).

An important implication of the present findings is that *Quercus persica* lozenges may represent a promising addition to the spectrum of gag reflex management strategies available to dental practitioners. While the current study demonstrated superiority over placebo, direct comparisons with established pharmacological approaches, such as topical lidocaine and benzydamine hydrochloride, as well as non-pharmacological methods including behavioral techniques, acupuncture, acupressure, and aromatherapy, were beyond the scope of this trial. Consequently, the present study provides a foundation for future comparative clinical investigations designed to evaluate relative efficacy, safety, patient acceptability, cost-effectiveness, and long-term outcomes across pharmacological and non-pharmacological interventions. Such studies would help define the optimal role of *Quercus persica* lozenges within evidence-based gag reflex management protocols.

## Conclusion

This randomized trial demonstrated that Quercus persica lozenges significantly reduce gag reflex severity in adult dental patients, particularly in the soft palate and tonsillar pillars. The lozenge was well tolerated, easy to administer, and showed a rapid localized effect. However, these findings should be interpreted cautiously due to the short-term, single-dose design and absence of comparative or patient-reported outcomes. Further research is warranted to assess long-term use, repeated dosing, direct comparisons with standard treatments (e.g., lidocaine spray), and mechanistic pathways. Future studies should also include patient-reported outcomes such as taste acceptability and satisfaction to inform clinical adoption.

## Data Availability

The data used in this study are available from the corresponding author on request.

## Abbreviations

GCP: Good Clinical Practice
